# A Laboratory Blizzard: Cold Agglutinin Disease Mimicking Tumor Lysis Syndrome

**DOI:** 10.51894/001c.165959

**Published:** 2026-07-31

**Authors:** Jash Mody, Michael Collins, David Moore, Myles Jerret

**Affiliations:** 1 Henry Ford - Wyandotte

## 104

Cold agglutinin disease (CAD) is an uncommon autoimmune hemolytic anemia that can produce profound laboratory artifacts, occasionally mimicking life-threatening metabolic emergencies such as tumor lysis syndrome (TLS). We report a woman with B-cell lymphoma and known CAD who was referred to the emergency department to “rule out TLS” after outpatient laboratories demonstrated severe hyperkalemia, anemia, and rising creatinine. Despite reported potassium values as high as 7–9 mmol/L, the patient was entirely asymptomatic with normal vital signs. Importantly, her electrocardiogram demonstrated normal sinus rhythm with normal intervals (QRS 86 ms, QTc 430 ms) and no peaked T waves, conduction delays, or repolarization abnormalities suggestive of hyperkalemia.

In the ED, repeated serum samples were flagged for hemolysis or clotting. Potassium values ranged from 7–9 mmol/L, hemoglobin appeared to decline from 10.2 to 8.3 g/dL over three hours, LDH was elevated in clotted specimens, and creatinine fluctuated from 0.75 to 1.17 mg/dL. Collectively, these abnormalities raised concern for TLS; however, the absence of hyperkalemia-associated EKG changes despite critically elevated reported potassium prompted reconsideration of the results. With true potassium levels of this magnitude, characteristic EKG findings would be expected.

A heparinized venous blood gas revealed a potassium of 4.3 mmol/L with hemoglobin consistent with baseline and no hemolysis. Saline replacement studies confirmed in vitro red blood cell agglutination. Repeat complete blood count and chemistries obtained using warmed, hand-carried specimens demonstrated stable hemoglobin and normal electrolytes. TLS was excluded by Cairo–Bishop criteria. The patient remained clinically stable and was discharged with warmth precautions and hematology follow-up.

CAD is mediated by IgM autoantibodies that bind red blood cells at low temperatures, causing agglutination and complement-mediated hemolysis. In vitro cooling can produce pseudohyperkalemia, spurious anemia, elevated LDH, apparent renal dysfunction, and clotted specimens. This case underscores the importance of correlating laboratory data with the clinical presentation. A normal EKG in the setting of reported severe hyperkalemia should prompt evaluation for pseudohyperkalemia. Recognition of CAD-related laboratory artifact prevents unnecessary intervention and reinforces a core emergency medicine principle: treat the patient, not the numbers.

